# The influence of physical exercise on negative emotions in adolescents: a meta-analysis

**DOI:** 10.3389/fpsyt.2024.1457931

**Published:** 2024-11-12

**Authors:** Tong Wang, Weicheng Li, Jiaxin Deng, Qiubo Zhang, Yongfeng Liu

**Affiliations:** School of Sports Training, Chengdu Sport University, Chengdu, Sichuan, China

**Keywords:** physical exercise, adolescents, negative emotions, mental health, meta-analysis

## Abstract

**Background:**

Adolescence is also accompanied by ongoing mood changes (relative to childhood and adulthood), which can trigger more extreme negative emotional responses. Physical exercise alleviates negative emotions and reduces the risk of mental illness. However, the effect of physical exercise on negative emotions in adolescents is unclear, so it is valuable to synthesize previous studies with meta-analysis.

**Objective:**

To examine the influence of physical exercise (PE) intervention on negative emotions in adolescents aged 10 to 19 years.

**Methods:**

We retrieved the articles from PubMed, Web of Science, EBSCO, Cochrane, and Embase up to April 11, 2024. The main search terms were physical exercise, negative emotions, adolescents, randomized controlled trials. The meta-analysis was conducted using Review Manager 5.3. A random-effects model was employed to calculate the standardized mean difference (SMD) and 95% confidence interval (CI). Subgroups were analysed as the type of negative emotions, type of control group, intervention type, duration, time, frequency.

**Results:**

The PE intervention group exhibited a significantly superior improvement in alleviating negative emotions compared to the control group (SMD = -0.59, 95% CI: -0.92 to -0.26, p < 0.01, Z = 3.50, I² = 95%). PE was particularly effective in mitigating adolescent depression (SMD = -0.67, 95% CI = -1.07 to -0.28, p < 0.01, I² = 96%) but did not yield significant results in reducing adolescent anxiety (SMD = -0.29, 95% CI = -0.63 to 0.05, p = 0.10, I² = 95%).

**Conclusion:**

PE intervention can ameliorate negative emotions in adolescents.

**Systematic Review Registration:**

https://www.crd.york.ac.uk/prospero/, identifier CRD42024534375.

## 1 Introduction

Negative emotions are generally indicative of distress and are often conceptualized in varying intensities, such as anxiety, depression, sadness, and anger (1). Among these, depression and anxiety are frequently employed as predictors (2). Depression is often a chronic and recurring condition (3) associated with high levels of psychological distress, impairments in functioning, and poor physical health (4). Depression is the most predominant aspect of negative affect and a major contributor to the global burden of disease in young people under the age of 25 (5). It is estimated that one in five people will be affected by depression in their lifetime, and the majority of people with depression begin to experience the onset of the illness in adolescence to young adulthood (6, 7). Anxiety is the most common manifestation of psychopathology in youth, negatively affecting academic, social, and adaptive functioning and increasing risk for mental health problems into adulthood (8, 9). Approximately 1 in 4 adolescents exhibits increased levels of worry and anxiety. In addition, adolescent anxiety predicts ongoing mental health problems throughout life (10). Therefore, depression and anxiety are the most important aspects of negative emotions, and this study also focuses on these two aspects of negative emotions.

Adolescence, a period characterized by rapid growth and maturation, neuronal plasticity (11, 12), identity formation (13), and the establishment of behavioral tendencies, plays a crucial role in shaping mental well-being, potentially steering it in either positive or negative directions (14, 15). During this critical phase, individuals experience significant physical, psychological, and social development, which makes them particularly susceptible to negative emotional states influenced by various factors. Adolescence brings profound changes in the social environment, physical growth, and dramatic hormonal changes (16, 17). Behaviorally, adolescence is also accompanied by constant emotional changes (relative to childhood and adulthood), which can trigger more extreme emotional responses for at-risk individuals (18). Numerous studies have revealed that the incidence of negative emotions escalates considerably during adolescence, surpassed only by behavioral disorders (19–21). The predominant clinical manifestations of negative emotions in adolescents encompass apathy, somatic complaints, impaired concentration, indecision, overwhelming guilt, reckless behavior, disinterest in food or compulsive overeating, resulting in significant weight fluctuations, memory lapses, fear of death, defiance, pervasive sadness, anxiety, or despair, nocturnal insomnia, and excessive daytime drowsiness (22). It is therefore not surprising that adolescence is also a time when emotional symptoms such as anxiety and depression are often present. These emotional states not only exacerbate psychological suffering and impair the mental health of adolescents but are also closely associated with deliberate or inadvertent harmful behaviors (15, 23), such as smoking (24), drinking (25), and other detrimental activities. Hence, it is logical to conclude that an adolescent’s environmental exposure could mitigate the development of negative emotions later in life (26).

Physical exercise (PE) encompasses aerobic activity, resistance training, as well as both physical and mental exercises (27). Numerous studies have revealed that engaging in physical activity not only bolsters mental health but also mitigates negative emotions and diminishes the risk of mental disorders (28, 29). For example, regular and appropriate PE has the potential to transform the brain’s structure and function, thereby ameliorating negative emotional states by increasing levels of dopamine, serotonin, and norepinephrine (30). Scholars have also established a correlation between adhering to the three recommended 24-hour activity guidelines and a reduced risk of depression and anxiety (31). Research conducted among school-aged children in China indicated that those who adhered to the 24-hour activity standards exhibited the lowest risk of experiencing negative emotions (32). According to findings from Wang’s study, a six-week regimen of PE has proven effective in alleviating symptoms of depression among adolescents aged 12 to 18 (27).

Research on negative emotions in adolescents is limited. Considering the influences noted in earlier studies and adolescents’ natural emotional sensitivity, alongside the positive impacts of PE interventions on their emotional well-being, our study aimed to investigate negative emotions in individuals aged 10 to 19 years. We conducted a meta-analysis of existing literature to examine how the duration, time, and frequency of PE interventions affect study outcomes. Based on this, the hypothesis of this study was formulated: This study hypothesizes that physical exercise interventions will significantly reduce symptoms of anxiety and depression in adolescents.

## 2 Methods

Following the guidelines outlined in the Preferred Reporting Items for Systematic Reviews and Meta-Analysis (PRISMA) (33) and the Cochrane Handbook for Systematic Reviews and Meta-Analysis (34), this review was conducted. Moreover, the protocol for this review was duly registered on PROSPERO under the registration number CRD42024534375.

### 2.1 Search strategy

For this study, we searched PubMed, Web of Science, EBSCO, Cochrane, and Embase up to April 11, 2024. The main search terms were physical exercise, negative emotions, adolescents, randomized controlled trials, etc., and the search terms were connected by AND and the search terms were connected by OR. The search strategy was a Boolean logic search with the following search strategies: (“physical activity” or “physical exercise” or “sports activities” or “physical education” or “sport movement”, sport or “athletic sports” or “aerobic exercise” or “aerobic training” or “resistance exercise”, “muscle-strengthening exercise” or “strength training”, “fitness game”) AND (“negative emotion” or anxiety or anxious or depression or depressive or depress or pressure or stress or “psychological ill-being” or “mental disease”) AND (adolescent or teenager or “junior high school students” or “senior high school students”) AND (“randomized controlled trial” or RCT). The details of our search terms are outlined in **Table 1**, demonstrating the meticulousness and accuracy of our research approach. The study was conducted independently by two researchers (TW and WCL), with a third researcher (YFL) consulted in case of disagreement. In this case, WCL performed the first stage of screening based on the title and abstract, and TW performed the second stage of screening by reading the full text. Finally, the data were analyzed by TW, WCL, and JXD and supervised and reviewed by QBZ and YFL.

**Table 1:** Summary of search terms.

Category		Included search terms
Physical exercise		(“physical activity" or "physical exercise" or "sports activities" or "physical education" or "sport movement", sport or "athletic sports" or "aerobic exercise" or "aerobic training" or "resistance exercise", "muscle-strengthening exercise" or “strength training", "fitness game")
	AND	
Negative emotion		("negative emotion" or anxiety or anxious or depression or depressive or depress or pressure or stress or "psychological ill-being" or "mental disease")
	AND	
Adolescent		(adolescent or teenager or "junior high school students" or "senior high school students")
	AND	
Randomized controlled trial		(“randomized controlled trial” or RCT)

### 2.2 Eligibility criteria

The relevant studies’ inclusion criteria were established following the PICOS framework. For participants (P), studies involving adolescents aged 10 to 19 years were considered. Regarding Intervention (I), the experimental group received various forms of PE intervention. Comparison (C) groups encompassed no-exercise (NT), wait-list (WL), and attention/activity placebo (AP) conditions. Outcome (O) measurements are primarily focused on assessing negative emotions in adolescents. Lastly, Study Design (S) adhered to the randomized controlled trial methodology.

The following criteria were applied to exclude relevant studies: literature not in English, including unpublished materials, theses, and reviews; studies involving adults, animals, or special populations; literature lacking valid data extraction; duplicate publications; and full texts that were inaccessible.

### 2.3 Data extraction

The study followed the PRISMA statement guidelines when extracting data and selecting studies. Duplicate studies were eliminated using EndNote 20 software to consolidate articles from each source into a unified database. Data extraction was mainly carried out independently by two researchers (TW and WCL), with a third researcher (YFL) being consulted in case of disagreement. Data were analyzed by TW, WCL, and JXD and supervised and reviewed by QBZ and YFL. Investigations into the attrition of treatment during follow-up were conducted independently by two researchers (TW and QBZ), who examined the included literature and consulted a third researcher (YFL) in cases of discordance. When data cannot be extracted, we will contact the author of the article to resolve the issue. If we cannot get in touch, we will extract the data using WebPlotDigitizer software.

Utilizing Review Manager 5.3 (35), data were inputted for both intervention and control groups, encompassing mean values, standard deviations, and participant counts. To accommodate the anticipated heterogeneity between trials attributed to the implementation of diverse PE interventions, meta-analysis pooling was conducted using a random effects model. To facilitate the aggregation of data from various negative emotions symptom scales, the effect was evaluated as the standardized mean difference (SMD), calculated using Hedges’ g, adjusted for small sample size bias, accompanied by 95% confidence intervals (CI) (36), and heterogeneity was evaluated through standard parameters of the I^2^ statistic (34). In cases where the test indicated substantial heterogeneity (I^2^ > 50%), we employed subgroup analysis and sensitivity analysis to elucidate the findings (37).

Subgroup analysis was utilized to investigate potential factors influencing the impact of PE on negative emotions. Pre-specified subgroup objectives were type of negative emotions (depression vs. anxiety), type of control group (WL/NT vs. AP), type of exercise intervention (aerobic vs. resistance vs. mixed), exercise intervention duration (<12 weeks vs. ≥12 weeks), exercise intervention time (<60min vs. ≥60min), exercise intervention frequency (≤3 times per week vs. >3 times per week).

### 2.4 Methodological quality assessment

The included studies were independently quality assessed using the Cochrane Risk of Bias Tool (38) by researchers TW and WCL, respectively. In this process, consensus is sought through in-depth discussion of any disagreements that arise in the assessment. If there were disagreements that could not be resolved through discussion, a third reviewer (CY) was consulted to ensure the objectivity and accuracy of the results. The methodological quality of the included studies was evaluated using the Cochrane risk of bias criteria, comprising seven items: random sequence generation (selection bias), allocation concealment (selection bias), blinding of participants and personnel (performance bias), blinding of outcome assessment (detection bias), incomplete outcome data (attrition bias), selective reporting (reporting bias), and other biases. Each item was assessed as “low risk”, “uncertain risk”, or “high risk” based on the responses to the signaling questions, contributing to an overall judgment of bias for each study assessed.

## 3 Results

### 3.1 Study selection

**Figure 1:**
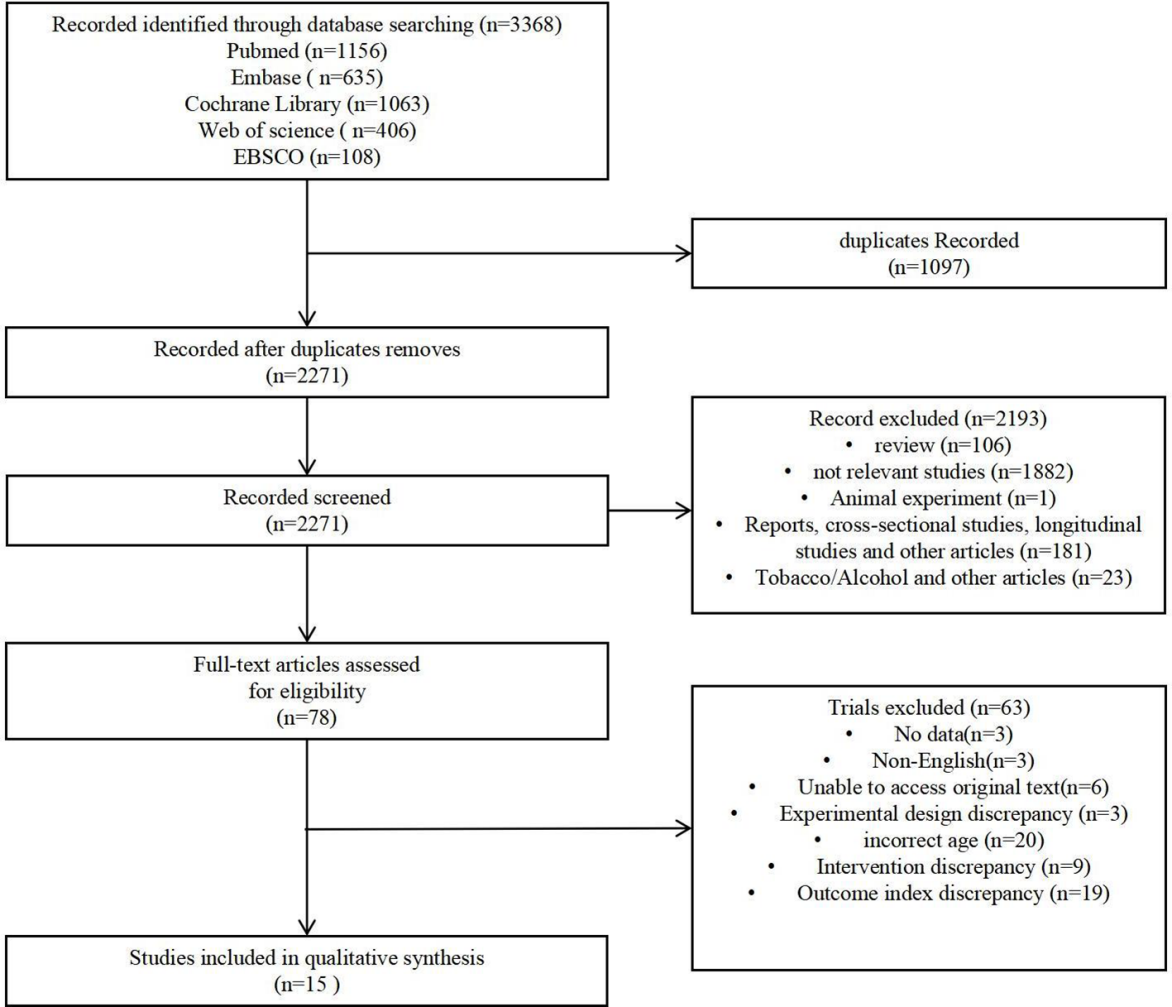
Flow chart of literature retrieval.

The literature search results and research selection process are shown in **Figure 1**. Initially, 3368 articles were retrieved from the databases, of which 1156 were from Pumbed, 635 from Embase, 1063 from Cochrane, 406 from Web of Science, and 108 from EBSCO. Following the removal of duplicates, 2271 studies remained, while 2193 studies did not meet the eligibility criteria during the title and abstract screening phases. These ineligible papers covered reviews (n = 106); not relevant studies (n = 1882); animal experiments (n = 1); reports, cross-sectional studies, longitudinal studies, and other articles (n = 181); and alcohol/tobacco and other articles (n = 23). Out of 78 studies, 63 were excluded after reading their full texts: no data (n = 3); non-English (n = 3); unable to access original text (n = 6); experimental design discrepancy (n = 3); incorrect age (n = 20); intervention discrepancy (n = 9); and outcome index discrepancy (n = 19). A total of 15 articles were eventually included in the meta-analysis.

### 3.2 Study characteristics

As illustrated in **Table 2**, the sample sizes of the trials ranged from 24 to 1066 participants, with mean ages spanning from 12 to 18.8 years. Two studies included subjects with a healthy mental status, seven studies included subjects with an unhealthy mental status, and six studies did not report the mental status of the included subjects. A summary of the characteristics of the PE interventions implemented in each trial is provided in **Tables 2** and **3**. The majority of trials employed aerobic exercise, though there was significant variation in the types of PE. The duration of the interventions varied between 6 to 48 weeks, with sessions occurring 1 to 5 times per week. The time commitment per session ranged from 20 to 440 minutes. The control groups consisted of NT (n = 3), WL (n = 3), and AP (n = 8). Ten studies used per-protocol analysis, and five studies used intention-to-treat analysis.

**Table 2:** Included trial characteristics.

Included studies	Sample size (Exp/Ctrl)	Age (Mean)	Mental state of the adolescents	Intervention	Duration (Weeks)	Time (Min)	Frequency (Times/Week)	Outcome	Outcome Measurement Tools	Control group	Mode of analysis
Ahmed, 2023 (39)	160/160	14.4	-	Aerobic	12	60	1	Depression	CESD-10	NT	Protocol
Carter, 2015 (40)	36/28	15.4	Unhealthy	Mixed	6	60	2	Depression	CDI-2	TAU	Intention-to-treat
Chung, 2021 (51)	115/113	12-16	Healthy	Adventure‐based training	-	2-day/1-night	-	Depression	CES-DC	AP	Protocol
Crews, 2004 (41)	34/32	-	-	Aerobic	6	20	3	Anxiety Depression	STAIC BDI	AP	Protocol
Goldfield, 2015 (42)	73/78/72/75	15.6	Healthy	Aerobic Resistance Mixed	22	20-45	4	Depression	BRUMS	NT	Protocol
Hughes, 2013 (43)	14/12	17	Unhealthy	Aerobic	12	30-40	2-3	Depression	CDRS-R	AP	Intention-to-treat
Jeong, 2005 (52)	20/20	16	Unhealthy	Aerobic	12	45	3	Depression	SCL-90-R	WL	Intention-to-treat
Khalsa, 2012 (44)	67/34	16.8	-	Aerobic	11	30-40	2-3	Anxiety Depression	POMS-SF	AP	Protocol
Lima, 2022 (45)	308/251/289/218	17	-	Aerobic	24	200/240/440	4	Depression	CES-D	AP	Protocol
Melnyk, 2015 (46)	9/9	16	Unhealthy	Aerobic	48	20	1	Depression	BYI-II	WL	Protocol
Nabkasorn, 2006 (47)	21/28	18.8	Unhealthy	Aerobic	8	50	5	Depression	CES-D	WL	Intention-to-treat
Noggle, 2012 (48)	36/15	17	-	Aerobic	10	30-40	2-3	Anxiety Depression	POMS-SF	AP	Protocol
Norris, 1992 (49)	14/15/16	16.7	-	Aerobic	10	25-30	2	Anxiety Depression	MAACL	AP	Protocol
Philippot, 2022 (50)	20/20	15	Unhealthy	Mixed	6	-	4	Anxiety Depression	HADS-A HADS-D	AP	Intention-to-treat
Roshan, 2011 (53)	12	16.9	Unhealthy	Aerobic	6	-	3	Depression	HAM-D	NT	Protocol

Exp, Experiment group; Ctrl, Control group; CESD-10, Center for Epidemiologic Studies Depression Scale; CDI-2, Children’s Depression Inventory-2; CES‐DC, Depression Scale for Children; STAIC-Trait, Anxiety Inventory for Children; BDI, Beck Depression Inventory; ERICA, Emotion Regulation Index for Children and Adolescents; HBSC, Health Behavior in School-aged Children; BRUMS, 24-item Brunell Mood Scale; CDRS-R, Childs Depression Rating Scale–Revised; Symptom Check List-90-Revision; POMS-SF, The Profile of Mood States short form POMS-SF; PSS, The Perceived Stress Scale; CES-D, Centre for Epidemiological Studies Depression scale; BYI-II, The depression subscale of the Beck Youth Inventory II; HADS, The Hamilton Depression Rating Scale; HAM-D, Hamilton Depression Rating Scale; AP, Attention/Activity Placebo; NT, No-Treatment Control; WL, Wait-List control; TAU, Treatment as Usual; -: No report.

**Table 3:** Subgroup analyses based on the primary meta-analysis.

Subgroup analysis	K	SMD	95%CI	*p* value	Heterogeneity	Test for subgroup difference
Primary meta-analysis	15	-0.59	-0.92 to -0.26	*p*<0.01	X^2^ = 458.54, df = 25 (*p*<0.00001), Z = 3.50, I^2^ = 95%	
Type of Negative Emotions (2 sub-group analyses)						
Depression	15	-0.67	-1.07 to -0.28	*p*<0.01	X^2^ = 445.61, df = 19 (*p*<0.00001), Z = 3.37, I^2^ = 96%	X^2^ = 2.10, df = 1 (P = 0.15), Z = 3.50, I^2^ = 52.5%
Anxiety	4	-0.29	-0.63 to 0.05	*P = 0.10*	X^2^ = 10.39, df = 5 (*p*<0.00001), Z = 1.66, I^2^ = 52%	
Type of Control Group (2 sub-group analyses)						
PE v. NT/WL	6	-1.37	-2.25 to -0.50	*p*<0.01	X^2^ = 193.78, df = 7 (*p*<0.00001), Z = 3.08, I^2^ = 96%	X^2^ = 6.38, df = 1 (P = 0.01), Z = 3.49, I^2^ = 84.3%
PE v. AP	8	-0.23	-0.38 to -0.08	*p*<0.01	X^2^ = 36.17, df = 16 (P=0.003), Z = 3.04, I^2^ = 56%	
Type of Exercise Intervention (3 sub-group analyses)						
Aerobic	10	-0.45	-0.68 to -0.21	*p*<0.01	X^2^ = 95.22, df = 17 (*p*<0.00001), Z = 3.77, I^2^ = 82%	X^2^ = 91.02,df = 2 (P<0.00001), Z = 3.78, I^2^ = 97.8%
Resistance	1	-2.49	-2.91 to -2.06	*p*<0.01	-	
Mixed	3	-0.17	-0.40 to 0.06	*P* = 0.15	X^2^ = 0.94, df = 3 (P = 0.81), Z = 1.43, I^2^ = 87%	
Duration (2 sub-group analyses)						
<12 weeks	8	-0.32	-0.54 to 0.10	*P* = 0.004	X^2^ = 424.31, df = 9 (P = 0.01), Z = 2.90, I^2^ = 52%	X^2^ = 3.55, df = 1 (P = 0.06), Z = 3.04, I^2^ = 71.8%
≥12 weeks	6	-0.97	-1.61 to 0.33	*P* = 0.003	X^2^ = 28.97, df = 14 (P = 0.003), Z = 2.97, I^2^ = 98%	
Time (2 sub-group analyses)						
<60min	9	-0.59	-0.98 to -0.20	*P* = 0.003	X^2^ = 141.4, df=16 (*p*<0.00001),Z=3.00, I^2^ = 89%	X^2^ = 0.05,df=1(P=0.83), Z=3.20, I^2^ = 0%
≥60min	3	-0.70	-1.56 to 0.16	*P* = 0.11	X^2^ = 294.69, df = 4 (*p*<0.00001), Z = 1.58, I^2^ = 99%	
Frequency (2 sub-group analyses)						
≤3times/week	11	-0.59	-1.10 to -0.08	*P* = 0.02	X^2^ = 256.98, df = 16 (*p*<0.00001),Z = 2.28, I^2^ = 94%	X^2^ = 0.00, df = 1 (P = 0.97), Z = 3.50, I^2^ = 0%
>3times/week	4	-0.58	-1.02 to -0.14	*P* = 0.01	X^2^ = 168.45, df = 8 (*p*<0.00001), Z = 2.56, I^2^ = 95%	

K, Number of trials; SMD, Standardized Mean Difference; CI, Confidence Interval; PE, Physical Exercise; NT, No-Treatment; AP, Attention/Activity Placebo; WL, Wait-List.

### 3.3 Risk of bias

**Figure 2:**
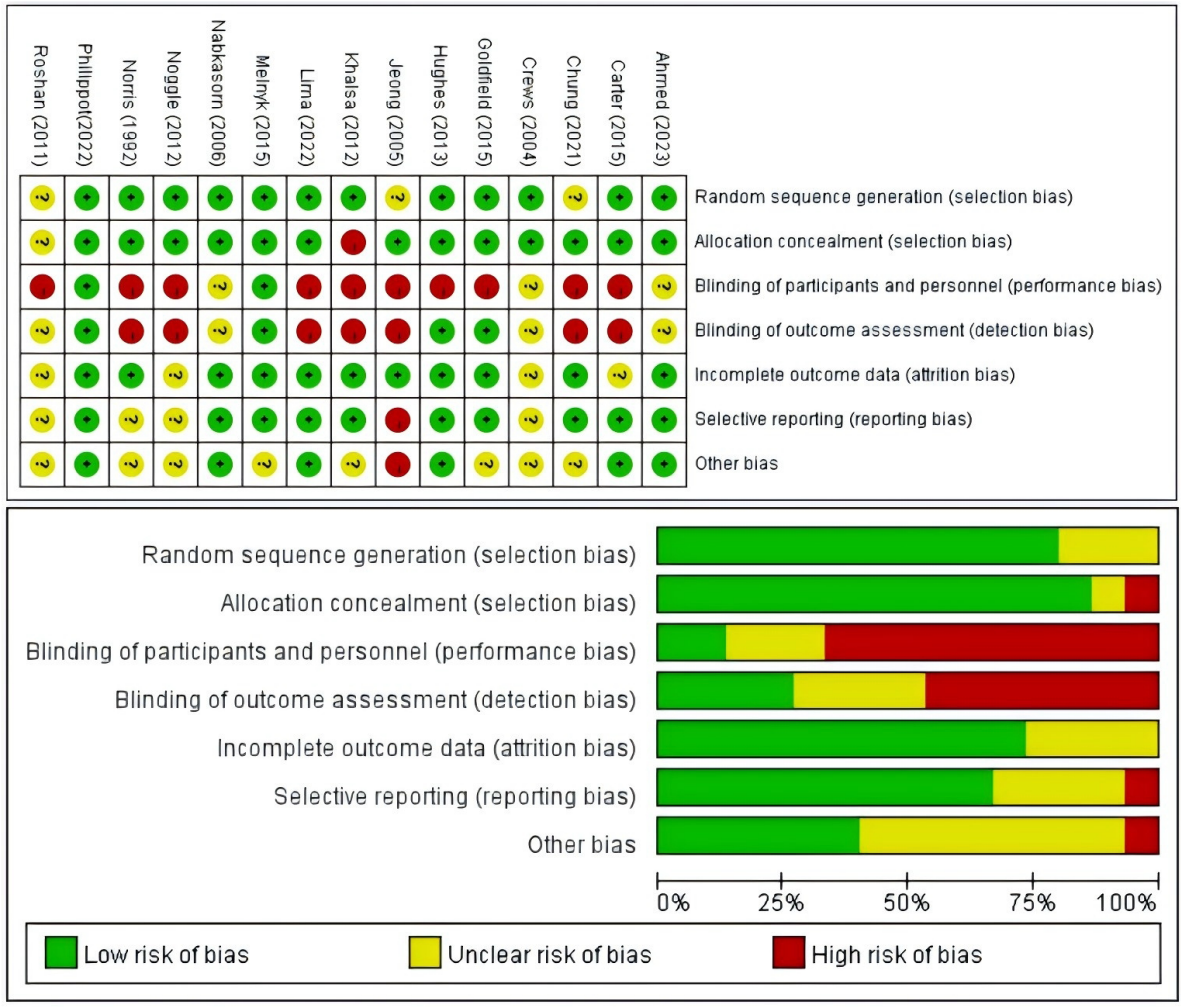
Results of the Cochrane risk of bias tool.

**Figure 3:**
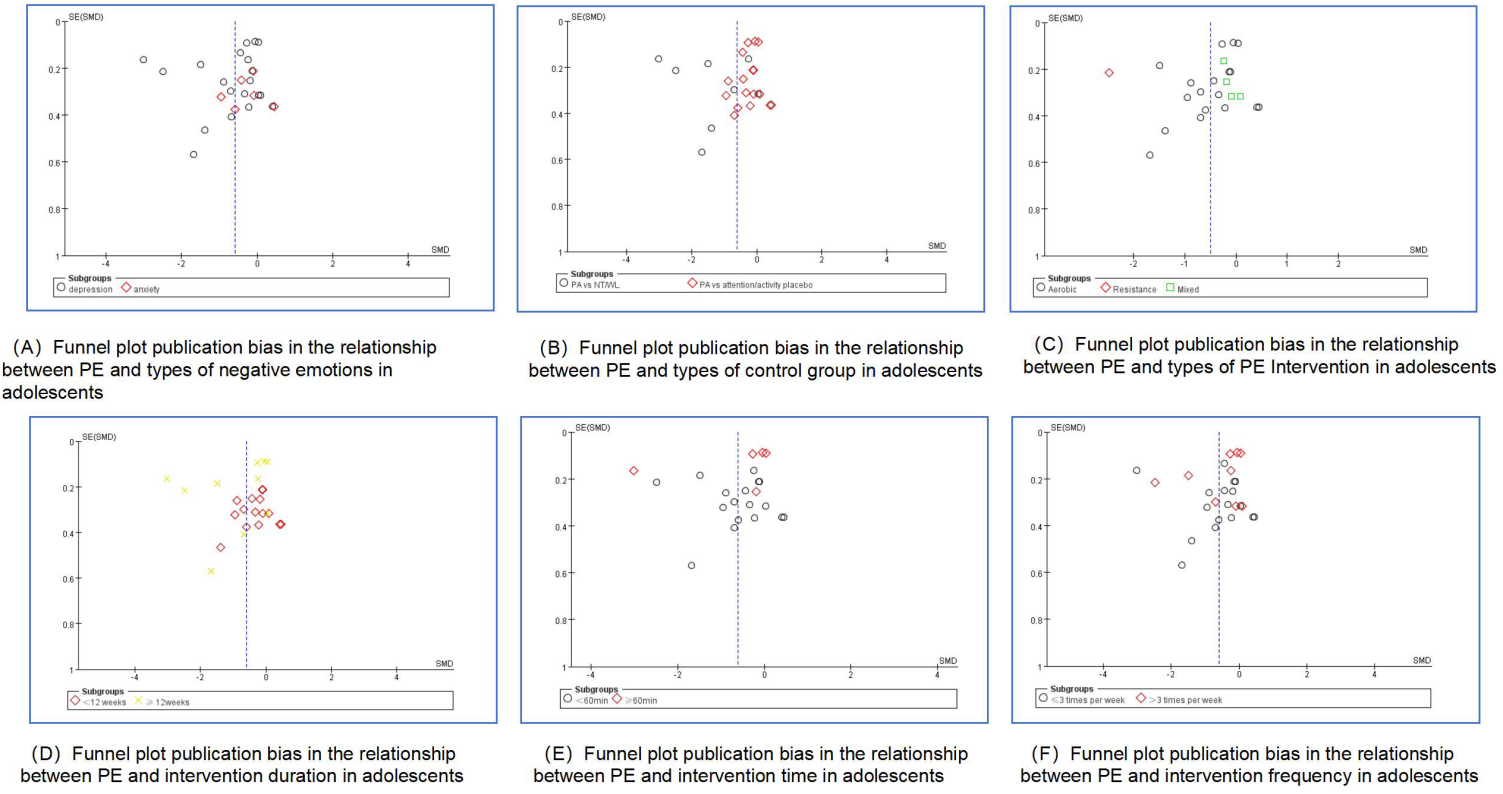
Funnel plot publication bias in the relationship between PE and negative emotions in adolescents (Subgroup analysis).

The methodological quality of the included literature was assessed using the Cochrane Risk Assessment Tool. For random sequence generation, 12 articles (39–50) were rated as low risk and 3 articles (51–53) as unclear risk. For allocation concealment, 13 articles (39–43, 45–52) were rated as low risk, 1 article (53) as unclear, and 1 article (44) as high risk. Regarding performance bias, 2 articles (46, 50) were rated as low risk, 3 articles (39, 41, 47) as unclear risk, and 10 articles (40, 42–45, 48, 49, 51–53) as high risk. Regarding detection bias, 4 articles (42, 43, 46, 50) were rated as low risk, 4 articles (39, 41, 47, 53) as unclear, and 7 articles (40, 44, 45, 48, 49, 51, 52) as high risk. For attrition bias, 11 articles (39, 42–47, 49–52) were rated as low risk and 4 articles (40, 41, 48, 53) as unclear risk. Regarding reporting bias, 10 articles (39, 40, 42–47, 50, 51) were rated as low risk, 4 articles (41, 48, 49, 53) as unclear risk, and 1 article (52) as high risk. Finally, with regard to other biases, 6 articles (39, 40, 43, 45, 47, 50) were rated as low risk, 8 articles (41, 42, 44, 46, 48, 49, 51, 53) as unclear risk, and 1 article (52) as high risk (**Figure 2**). The assessment of publication bias in the included studies was performed visually through an analysis of the funnel plot (**Figure 3**, **Supplementary Figure 1**). The funnel plot analysis reveals notable asymmetry, indicating potential publication bias. This asymmetry suggests that smaller studies with non-significant results may be underrepresented, consequently inflating the true effect size in our meta-analysis. Beyond publication bias, discrepancies in study design, including variations in sample size, methodology, and population characteristics, may also contribute to the observed asymmetry. Smaller studies often exhibit more variable effect sizes, further compounding the asymmetry.

### 3.4 Meta-analysis results

In evaluating the effects of PE on negative emotions in adolescents, fifteen studies utilized negative emotions, such as depression or anxiety, as outcome measures. Initially, these studies were tested for heterogeneity, revealing substantial differences among them (I² = 95% > 50%, p < 0.01). A high degree of heterogeneity (I² = 95%) in a meta-analysis indicates substantial variability across studies, likely due to differences in study populations, methodologies, contexts, or statistical issues. This variability can arise from factors like demographic differences, variations in interventions, study settings, and potential biases in publication. The implications are significant, as it suggests that the pooled results may not accurately reflect the true effect, limiting the generalizability of the findings. Therefore, we used a random-effects model and a way to minimize heterogeneity by dividing the subgroups. The meta-analysis, depicted in **Supplementary Figure 2**, demonstrated a significant improvement in negative affect in the PE intervention group compared to the control group (SMD = -0.59, 95% CI: -0.92 to -0.26, p < 0.01, Z = 3.50, I² = 95%). This indicates a notable reduction in negative emotions among adolescents who engaged in PE.

### 3.5 Sensitivity analysis

Sensitivity analyses were conducted to further explore the sources of heterogeneity. The process involved systematically excluding individual studies from the analysis one at a time to assess their impact on the overall results. Factors such as study design, sample size, and quality scores were considered in these analyses to determine their potential influence on heterogeneity. The results obtained after each exclusion were consistent with the initial analyses, indicating that no single study significantly affected the composite results. This consistency suggests that the composite effect size in this study is stable and robust.

### 3.6 Subgroup analysis

To explore potential modifications of PE effects on negative emotions, a subgroup analysis (**Table 3**) was conducted to assess the influence of specific factors. We divided the subgroups according to the Physical Activity Guidelines for Americans (54), a document that requires that the majority of the 60 minutes or more per day for children and adolescents should be moderate-intensity or vigorous-intensity aerobic exercise and should include vigorous-intensity physical activity at least 3 days per week. Therefore, the objectives of the subgroup analysis in this study were to determine the type of negative emotions (depression, anxiety), type of control group (WL/NT, AP), intervention type (aerobic, resistance, mixed), exercise intervention duration (<12 weeks, ≥12 weeks), intervention time (<60min, ≥60min), intervention frequency (≤3 times/week, >3 times/week).

#### 3.6.1 Types of negative emotions

**Figure 4:**
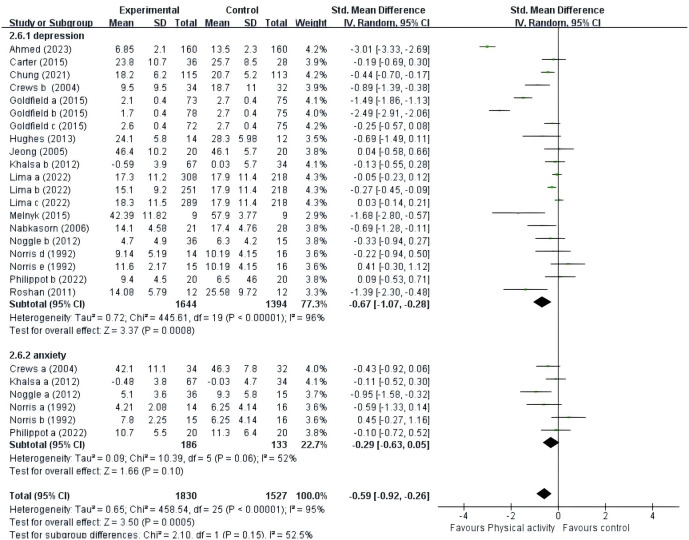
Forest plot of a meta-analysis of the relationship between PE and negative emotions after the intervention.

A total of 15 studies were included (**Figure 4**). 15 studies (39–53) provided data on the effect of PE intervention on adolescent depression; 4 studies (41, 44, 48–50) provided data on the effect of PE intervention on adolescent anxiety. A random-effects model was employed for the meta-analysis, revealing that the PE intervention had a pronounced impact on adolescent depression (SMD = -0.67, 95% CI = -1.07 to -0.28, p < 0.01, I² = 96%), whereas its effect on adolescent anxiety (SMD = -0.29, 95% CI = -0.63 to 0.02, p = 0.05, I² = 52%) was not statistically significant.

#### 3.6.2 Types of control groups

**Figure 5:**
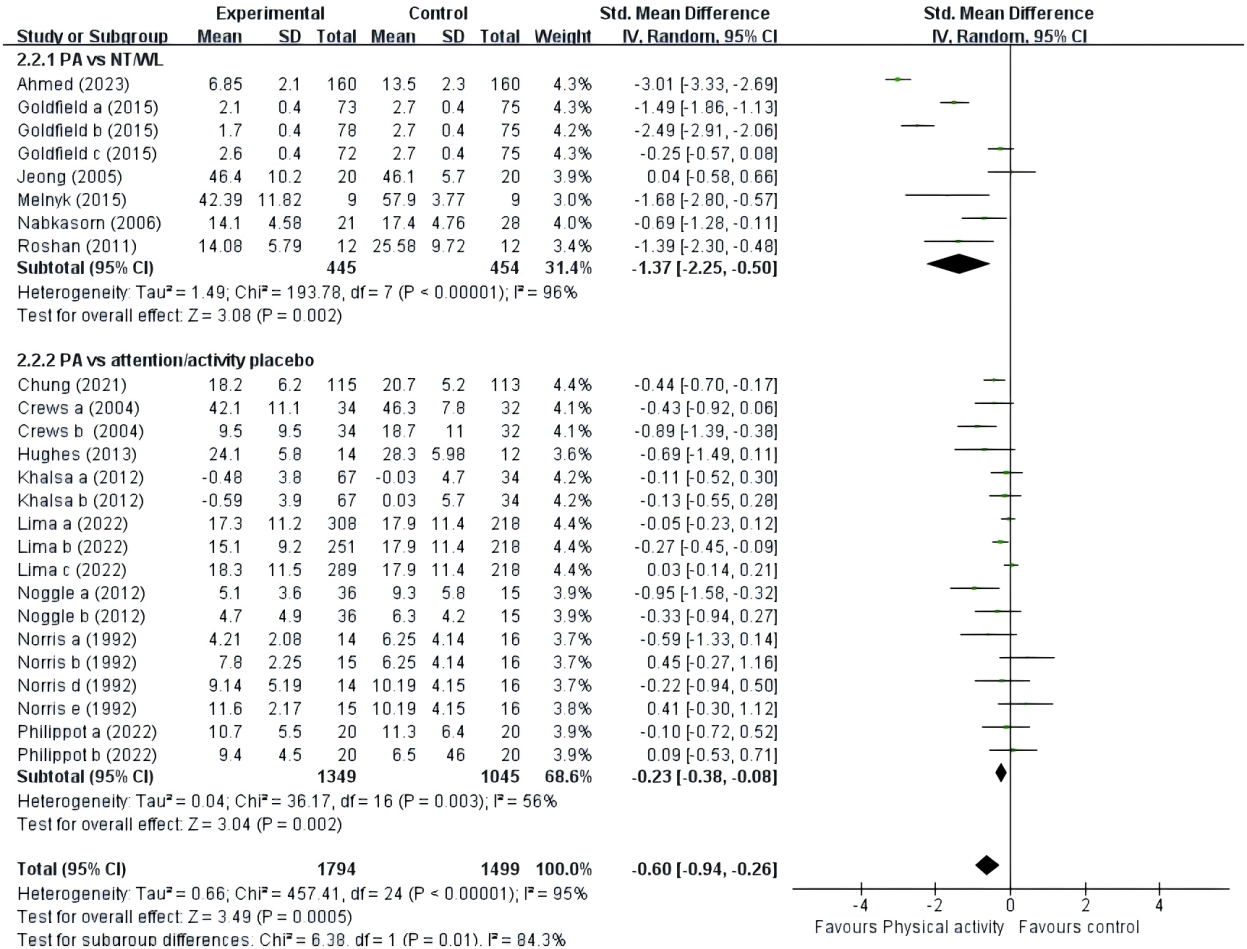
Forest plot of a meta-analysis of the relationship between PE and types of control group after the intervention.

A total of 14 studies were included (**Figure 5**). Six studies (39, 42, 46, 47, 52, 53) provided data on the type of control group that was the NT or WL group, and eight studies (41, 43–45, 48–51) provided data on the type of control group that was the placebo group. The results showed that PE improved negative emotions significantly in both the NT/WL (SMD = -1.37, 95% CI = -2.25 ~ -0.50, p<0.01, I^2^ = 96%) group and the AP group (SMD = -0.23, 95% CI = -0.38 ~ -0.08, p<0.01, I^2^ = 56%).

#### 3.6.3 Types of PE Intervention

**Figure 6:**
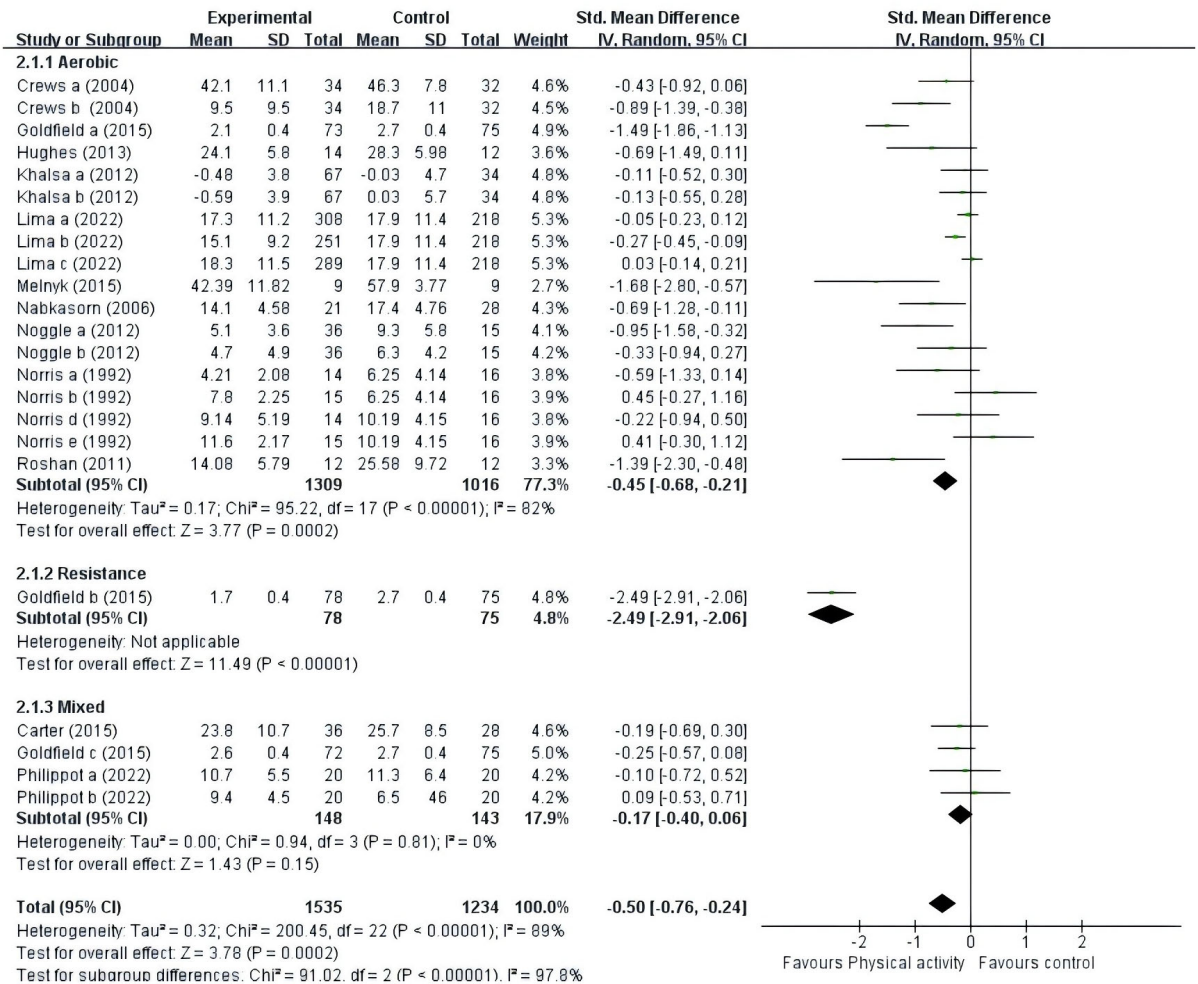
Forest plot of a meta-analysis of the relationship between type of PE intervention and negative emotions after the intervention.

A total of fourteen studies were included (**Figure 6**). Ten studies (41–49, 53) provided data for aerobic exercise; one study (42) provided data for resistance exercise; and three studies (40, 42, 50) provided data for mixed exercise. Subgroup analyses showed that aerobic exercise (SMD = -0.45, 95% CI = -0.68 ~ -0.21, p < 0.01, I^2^ = 82%) and resistance exercise (p < 0.01) significantly improved negative mood. However, mixed exercise (SMD = -0.17, 95% CI = -0.40 ~ -0.06, p = 0.15, I^2^ = 0%) was not significantly associated with negative mood in adolescents. Only one article on resistance exercise intervention was included, so the results should be interpreted with caution. Therefore, aerobic exercise had a significant effect on the improvement of negative mood in adolescents.

#### 3.6.4 PE intervention duration

**Figure 7:**
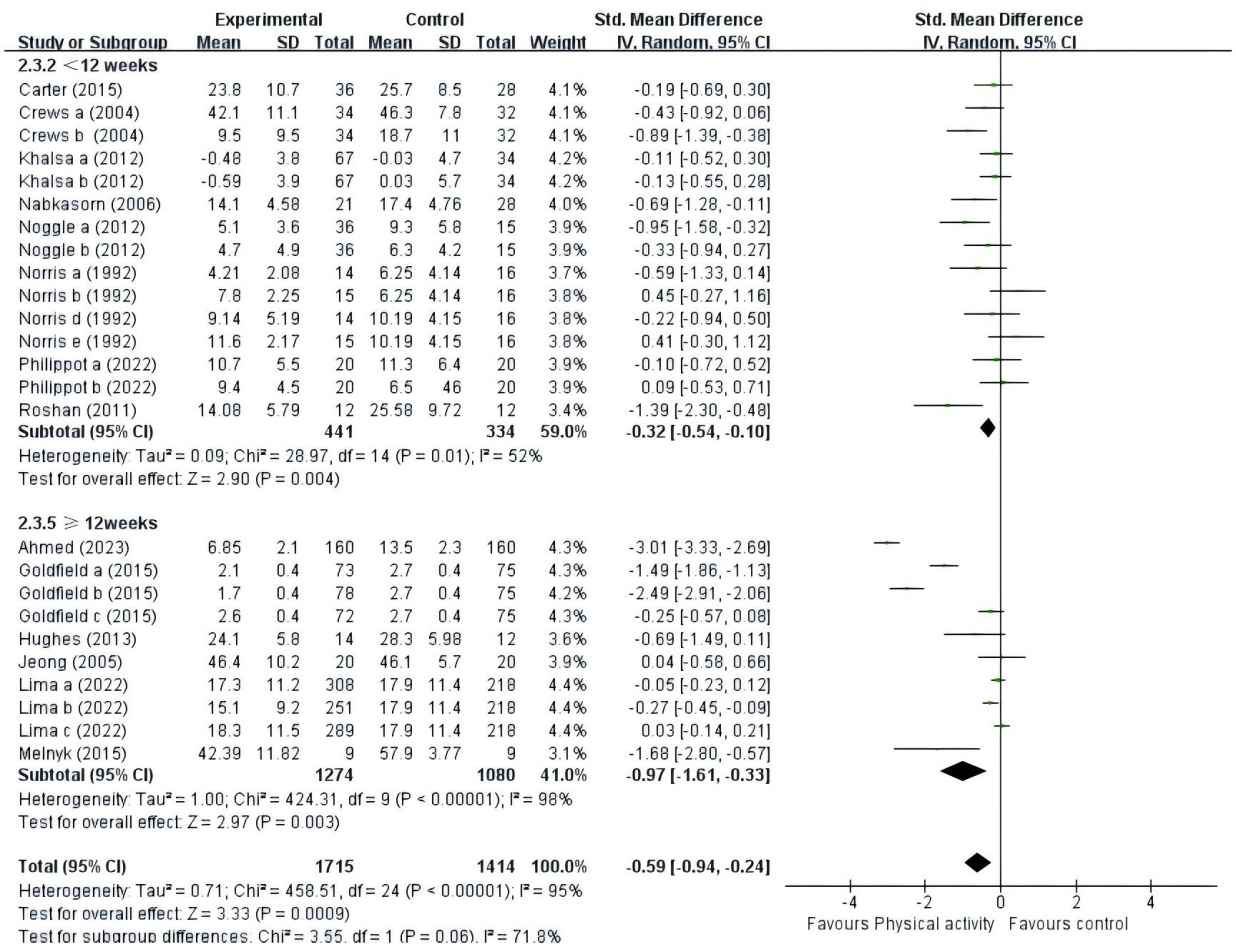
Forest plot of a meta-analysis of the relationship between PE intervention duration and negative emotions after the intervention.

A total of fourteen studies were included (**Figure 7**). Eight studies (40, 41, 44, 47–50, 53) provided data for intervention duration of less than 12 weeks; six studies (39, 42, 43, 45, 46, 52) provided data for intervention duration of at least 12 weeks. The results showed that PE interventions of less than 12 weeks (SMD = -0.32, 95% CI = -0.54 ~ -0.10, p = 0.004, I^2^ = 52%) or at least 12 weeks (SMD = -0.97, 95% CI = -1.61~-0.33, p = 0.003, I^2^ = 98%) had a significant effect on adolescents’ negative emotions. Overall, PE interventions lasting at least 12 weeks were significantly more effective in adolescents (p = 0.003) compared to those conducted for less than 12 weeks (p = 0.004).

#### 3.6.5 PE intervention time

**Figure 8:**
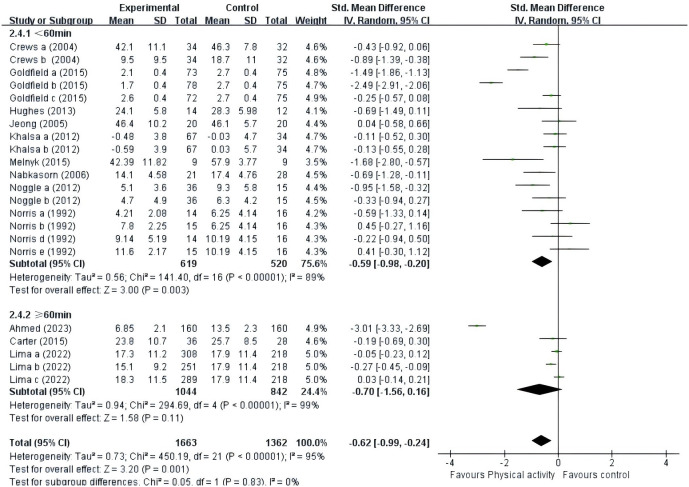
Forest plot of a meta-analysis of the relationship between PE intervention time and negative emotions after the intervention.

Twelve studies were encompassed in the analysis (**Figure 8**). Nine studies (41–44, 46–49, 52) provided data for intervention time less than 60 minutes; and three studies (39, 40, 45) provided data for intervention time greater than or equal to 60 minutes. The results showed that when the time was less than 60 minutes (SMD = -0.59, 95% CI = -0.98 ~ -0.20, p<0.01, I^2^ = 89%), the PE intervention had a significant effect on the negative emotions of adolescents. When time was at least 60 minutes (SMD = -0.70, 95% CI = -1.56 ~ 0.16, p = 0.11, I^2^ = 99%), PE intervention had no significant effect on adolescents’ negative emotions. Therefore, the PE of adolescents can be controlled in under 60 minutes.

#### 3.6.6 PE intervention frequency

**Figure 9:**
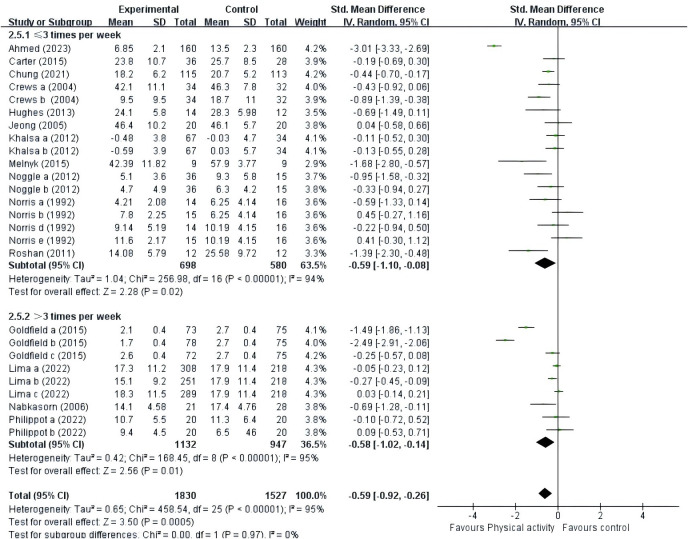
Forest plot of a meta-analysis of the relationship between PE intervention frequency and negative emotions after the intervention.

A total of fifteen studies were included (**Figure 9**). Eleven studies (39–41, 43, 44, 46, 48, 49, 51–53) provided data at a frequency of three or fewer times per week; and four studies (42, 45, 47, 50) provided data at a frequency of more than three times per week. The results showed that adolescents were better able to improve negative affect with both PE interventions less than three times per week (SMD = -0.59, 95% CI = -1.10 ~ -0.08, P = 0.02, I^2^ = 94%) and PE interventions more than three times per week (SMD = -0.58, 95% CI = -1.02 ~ -0.14, P = 0.01, I^2^ = 95%). Overall, the PE interventions conducted with adolescents more than three times per week (p = 0.01) proved to be more efficacious than those administered fewer than three times per week (p = 0.02).

## 4 Discussion

This study aimed to explore the impact of PE on adolescents’ experiences of negative emotions. From an initial pool of 3,368 search records, 15 studies were selected for inclusion in the meta-analysis. It was observed that since the included studies were randomized controlled trials focusing on PE interventions, the implementation of complete blinding was not feasible. Consequently, trials without blinding were not classified as low-quality during the literature quality assessment, as such a classification would be unjustified. The findings of this study indicate a significant reduction in adolescents’ negative emotions as a result of engaging in PE. The conclusions from a 2022 review by Hale and colleagues on the correlation between exercise and mental well-being corroborate this outcome. Further subgroup analyses revealed that adolescents who participated in aerobic exercise sessions lasting at least 12 weeks, conducted more than three times per week, and with each session lasting less than 60 minutes, experienced substantial improvements in negative emotions.

### 4.1 The effect of PE intervention on different kinds of negative emotions in adolescents

The results of our meta-analysis indicated that participation in sports was effective in alleviating depression but not anxiety in adolescents. First, the aspect of physical activity in alleviating depressed mood in adolescents agrees with previous studies (55, 56). Participation in exercise enhances the synaptic transmission of monoamines from a physiological perspective, thereby stimulating the release of endorphins (57). These substances exert inhibitory effects on the central nervous system, effectively diminishing discomfort and elevating the brain’s active state. Consequently, this process leads to a natural improvement in mood following physical exertion (58). In a comprehensive review conducted in 2001, Sallis examined 108 studies on PE and its effects on depression in children and adolescents. The review thoroughly documented the efficacy of PE in alleviating depressive symptoms in this demographic (55). A recent clinical study, notable for its integration of psychiatric and cardiological methodologies and compelling argumentation, conducted an exhaustive examination of 210 patients diagnosed with depression. The study’s findings revealed a significant correlation between elevated depressive symptoms and decreased levels of PE among participants (56). Our meta-analysis further corroborated the effectiveness of PE in mitigating depressive symptoms among adolescents. However, our study did not confirm that physical activity was effective in reducing anxiety (p = 0.10), which is inconsistent with previous studies that have examined the ability of physical activity to reduce anxiety. A study by Tao (59) illuminates this issue, focusing on visually impaired children, revealing that continuous engagement in physical activities and the reduction of prolonged sedentary behavior not only alleviated anxiety but also significantly contributed to its reduction (59). This discovery is supported by Gehricke’s research, thus bolstering the evidence surrounding the beneficial impact of PE on reducing anxiety (60). This uncertainty was particularly evident in 7 of the 12 studies, which accounted for approximately 58.3% of the research in this area (61). From our analysis, the PE intervention did not reduce anxiety, which is more consistent with some studies. Related studies also lacked sufficient evidence that PE reduces anxiety (40, 44, 49, 50). There are several possible reasons for this situation: Critical factors encompass sample size and diversity, which significantly influence the generalizability of findings. The intervention’s intensity, duration, and consistency are paramount, as low-intensity or short-duration exercises may fail to yield meaningful changes while inconsistent adherence can further attenuate the effects. The precision, reliability, and timing of measurement tools are also essential, as inadequately selected instruments or poorly timed assessments may overlook the intervention’s true impact. Psychological elements, such as baseline anxiety levels and placebo effects, may additionally sway outcomes. Furthermore, statistical and external considerations, including the choice of control groups and environmental variables, add layers of complexity to the interpretation. Future research should incorporate larger, more heterogeneous samples and interventions that are meticulously tailored to the specific characteristics of the target population.

### 4.2 The effect of types of control groups on adolescents’ negative emotions

This study investigated whether the type of control group affects the impact of physical exercise (PE) on adolescents’ negative emotions. Our meta-analysis showed that physical activity resulted in a slight reduction in negative emotions compared to the AP group (SMD = -0.23). Physical activity significantly reduced negative affect compared to the NT/WL group. The smaller improvement in the placebo group suggests that the placebo itself can provide some psychological benefit, but not as much as the actual physical activity. This highlights the importance of considering the placebo effect in psychological and physical interventions. A narrative review indicates that the mental health benefits of acute exercise may be due to placebo effects, as the acute psychological outcomes of exercise are not shown to be mediated by stimulus characteristics such as exercise duration or intensity (62). Philippot's (50) longitudinal HADS-A scores showed a reduction in anxiety symptoms over time in both groups. Thus, we can infer that participants benefited from the anxiolytic effects of the general psychiatric environment but did not derive added value from physical activity or did not have a sufficient effect size to reach significance, perhaps because of the placebo group (50). However, further research is necessary to substantiate this finding due to the observational nature of the analysis, the limited number of trials, and their low quality.

### 4.3 The effect of types of intervention on adolescents’ negative emotions

The study examined whether the type of intervention affected the impact of PE on adolescents’ negative emotions. Aerobic exercise emerged as the main focus in studies utilizing PE to alleviate adolescents’ negative emotions, highlighting the widespread adoption of aerobic exercise as a preferred intervention method, consistent with previous research (54, 63, 64). Involving the voluntary movement of skeletal muscles, aerobic exercise surpasses basal metabolic rates. This form of exercise is intricately linked with various facets of well-being, encompassing physical health, mental well-being, and overall life satisfaction (63). The elevation in heart rate and oxygen consumption induced by aerobic exercise triggers the release of endorphins and various other neurotransmitters renowned for their mood-enhancing properties. In the domain of mental health and exercise physiology, the hypothesis regarding the role of endorphins enjoys substantial support. Many researchers commonly believe that endorphins play a crucial role in conserving energy during physical exertion, potentially resulting in psychological benefits such as improved mood and reduced anxiety levels (65). Direct evidence also suggests that PE has the capacity to elevate plasma endorphin levels (66). In a comprehensive review conducted in 2021, Song found that university students experienced substantial alleviation from symptoms of depression through the efficacy of both aerobic exercise and traditional Chinese exercises (64). Only one study in our meta-analysis explored the impact of resistance exercise on negative emotions in adolescents (42). This emphasizes the importance of embracing all forms of PE to optimize well-being and emotional health in this demographic. Furthermore, despite the potential for comprehensive exercise to provide a range of physical benefits, such as increased strength, flexibility, and coordination, its effect on negative emotions may not consistently demonstrate significance (40, 42, 50). These findings underscore the critical role of tailoring interventions to align with individual preferences and needs, taking into account factors such as adherence and enjoyment, which are known to influence the effectiveness of PE interventions in enhancing mental well-being and alleviating negative emotions. Additionally, the limited inclusion of only three studies may have contributed to the perception that the observed effect lacks significance.

### 4.4 The effect of PE intervention duration on adolescents’ negative emotions

We assessed whether the duration of the intervention influenced the impact of PE on adolescents’ negative emotions. For this study, we categorized the included studies into two subgroups based on intervention duration: fewer than 12 weeks and at least 12 weeks. Our subgroup analysis revealed that PE interventions lasting fewer than 12 weeks or at least 12 weeks significantly affected adolescents’ negative emotions. Overall, interventions that lasted at least 12 weeks were more effective than those that lasted less than 12 weeks, which is consistent with previous studies (67–69). PE of a sustained and appropriate duration has the potential to induce significant changes in both the structure and function of the brain. These changes, in turn, have been associated with improvements in states of negative emotion. This improvement is attributed to heightened concentrations of dopamine, serotonin, and norepinephrine in the brain, known to positively influence mood regulation (30). However, long-term PE interventions may also lead to negative emotions not achieving that tan, possibly due to natural degradation processes, physical exhaustion, environmental monotony, or a combination of these factors (70, 71). Given the limited number of studies included, effect size statistics may be influenced, necessitating future large-scale studies to confirm the efficacy of PE intervention duration.

### 4.5 The effect of PE intervention time on adolescents’ negative emotions

In our meta-analysis, we examined the influence of intervention duration on the correlation between PE and adolescent negative emotions. The studies included were divided into two categories based on duration: those lasting less than 60 minutes and those lasting 60 minutes or more. Our subgroup analyses indicated that PE interventions lasting less than 60 minutes significantly enhanced adolescents’ negative emotions, whereas interventions lasting 60 minutes or more did not significantly affect these emotions, consistent with previous findings (54, 72, 73). Numerous studies have indicated that individuals with depression can alleviate negative emotions by engaging in aerobic exercises lasting 20 to 40 minutes (74). A separate retrospective analysis was conducted using data from a longitudinal study spanning a decade. This analysis revealed a significant correlation between regular PE, even for durations as short as 15 minutes, and reduced susceptibility to depression (73). The findings of this study align with the prevailing recommendations outlined in both the general health promotion guidelines of the Canadian government and those of the American Academy of Pediatrics. These guidelines emphasize the importance of engaging in at least 60 minutes of moderate-to-vigorous PE daily for children and adolescents to effectively maintain their physical and mental well-being. This highlights the critical role of consistent PE in promoting overall health and underscores the importance of adhering to such guidelines for optimal health outcomes in young individuals (75). However, based on the findings of this study, prolonged engagement may potentially lead to physical exhaustion, mental fatigue, or ennui among adolescents. Consequently, this could diminish the immediate mood-elevating effects commonly associated with PE and increase the likelihood of experiencing negative emotions. Extended periods of PE can stimulate androgen production akin to that observed with anabolic steroids, potentially resulting in a significant rise in irritability and aggression (76). Introducing novelty and variety into brief workout routines has been demonstrated to heighten enjoyment and intrinsic motivation. This enhancement of positive mood and emotion is reinforced by the diverse nature of these exercises, thereby aiding in the alleviation of negative emotions and contributing to overall emotional well-being.

### 4.6 The effect of PE intervention frequency on adolescents’ negative emotions

The study aimed to evaluate how intervention frequency affects the relationship between PE and negative emotions in adolescents. This meta-analysis categorized the studies into two groups based on intervention frequency: less than three times per week and three times per week or more. The subgroup analysis revealed that adolescents experienced enhanced improvement in negative affect with both less frequent (less than 3 times per week) and more frequent (3 times per week or more) PE interventions. Overall, PE interventions more than three times per week were more effective than interventions less than three times per week, which is consistent with previous research (54, 73, 77). Current clinical recommendations propose that to alleviate negative emotions in children, engaging in exercise sessions lasting at least 45 minutes on a minimum of three days per week is advisable. These guidelines highlight the importance of exercise frequency in alleviating negative emotions among children (77). Adolescents should engage in PE sessions at least three times weekly, as recommended by current clinical guidelines. This structured regimen facilitates the establishment of consistent exercise habits among adolescents. Regular participation in PE over time promotes positive physical and mental adaptations, contributing significantly to overall well-being and long-term adherence to exercise routines. Integrating PE into their weekly schedule can lead to sustained improvements in mood and emotional well-being. Moreover, maintaining a high frequency of PE sessions helps sustain the mood-enhancing benefits of exercise throughout the week. By spacing out their PE sessions and consistently exposing themselves to the positive physical effects of PE, adolescents can experience more enduring improvements in mood, fostering emotional stability and long-term well-being. Therefore, increasing the frequency of PE sessions to three or more times weekly represents a potentially effective strategy to enhance mental health and emotional well-being in adolescents. Further research should explore optimizing PE interventions tailored to the specific needs and preferences of adolescents.

### 4.7 Proposed clinical interventions based on meta-analysis findings

To translate the findings of the meta-analysis into effective clinical practices for mitigating negative emotions in adolescents, several intervention strategies can be proposed, each meticulously tailored to address the unique needs of this population. Among the most impactful approaches is the introduction of structured physical activity programs, which could be implemented in schools or community centers specifically targeting adolescents who grapple with negative emotions. Ideally, these programs should be conducted three to five times per week, with sessions lasting between 30 and 60 minutes at a moderate to vigorous intensity, contingent upon the individual’s fitness level and psychological state. The potential benefits of such programs are considerable, including the alleviation of symptoms associated with depression, anxiety, and stress, as well as enhancements in overall mood through the release of endorphins and the promotion of neuroplasticity. Moreover, group activities within these programs can provide essential social support, which is particularly advantageous for adolescents navigating negative emotions. However, challenges such as maintaining consistent participation, ensuring accessibility to facilities, and the necessity of individualized programming to accommodate varying fitness levels and emotional states must be carefully managed to optimize the success of these interventions.

Incorporating physical activity into existing therapeutic practices, such as cognitive-behavioral therapy (CBT), represents another promising intervention. This approach could involve the integration of brief exercise breaks—five to ten minutes of aerobic exercises—within therapy sessions or prescribing personalized exercise routines as part of therapy homework. The inclusion of physical activity in this context may enhance the overall efficacy of psychological treatments, empowering adolescents by equipping them with coping skills through movement. Nevertheless, this approach does pose challenges, including the need for additional training for therapists to integrate exercise effectively, as well as potential resistance from adolescents who may have low motivation or negative associations with physical activity.

Lastly, the development of school-based wellness programs that amalgamate physical education, mental health education, and mindfulness practices presents a holistic approach to addressing negative emotions in adolescents. These programs should incorporate curricula that educate students on the psychological benefits of exercise and stress management techniques, alongside peer support groups that encourage participation in physical activities and offer emotional support. The key advantages of this approach include the promotion of both physical and mental well-being and the cultivation of a culture that recognizes the interconnectedness of emotional health and physical activity. Nevertheless, the successful implementation of such programs necessitates coordination among educators, school counselors, and physical education instructors, and they must be designed to be sustainable and adaptable to the evolving needs of students.

In conclusion, the application of these meta-analysis findings offers a promising avenue for supporting adolescents with negative emotions through physical activity-based interventions. Whether through structured programs, integration into therapy, or comprehensive school-based initiatives, these strategies can be tailored to individual and group needs, though challenges such as adherence, accessibility, and the need for specialized training must be addressed. Future research should focus on further refining these interventions, identifying the most effective types and intensities of physical activity for different adolescent subgroups, and exploring innovative solutions to overcome the practical challenges in clinical applications.

## 5 Limitations of the review

Firstly, the limited sample size and the attrition of some participants during follow-up may not accurately represent all groups, resulting in biased outcomes. Consequently, future research should increase the sample size to enhance the robustness of subgroup analyses. Secondly, regarding data analysis, some subgroup analyses failed to yield reliable conclusions or were not conducted (e.g., intervention intensity) due to constraints in the number and characteristics of included studies. This resulted in significant heterogeneity among subgroups, obscuring crucial implications and limiting the generalizability of the findings. Thirdly, negative affect extends beyond depression and anxiety to encompass emotions such as stress, distress, sadness, and anger. The focus of this meta-analysis on depression and anxiety underscores the necessity for future research to investigate and address other forms of negative emotions. Finally, some results rely heavily on participants’ self-reported data. Self-reported behaviors may be subject to individual cognitive and memory biases, potentially affecting the accuracy of results and introducing reporting bias. Subsequent research efforts should explore the use of more objective measurement tools to minimize such biases.

## 6 Conclusion

The purpose of this study was to review the current literature on the relationship between negative emotions and physical activity in adolescents. The group engaged in physical activity exhibited significant improvement in ameliorating negative mood compared to the control group. Subgroup findings indicate that engaging in aerobic exercise for a minimum of 12 weeks, exceeding thrice weekly, with sessions not exceeding 60 minutes, proves highly efficacious. However, several limitations remain, such as smaller sample sizes, publication bias, the presence of heterogeneity in subgroup analyses, and the lack of objective measurements in measurement tools. Moreover, many of the studies in the meta-analysis were difficult to interpret because the study protocols were not standardized, especially not including the range of competence of the cohort, the duration of the study, the recommended exercise, and the monitoring of the control group. In addition, the variability of the results has to be considered, and the results of this study should be interpreted with caution. Considering the ability of physical activity to alleviate negative emotions among adolescents, there is a need to further explore the effects of physical interventions on alleviating adolescents’ negative emotions. Follow-up studies should thoroughly examine differences in such interventions in terms of gender, age group, and exercise intensity. In addition, it would be desirable to incorporate local control variables into physical activity programs and to juxtapose the results with international studies to assess the impact of physical activity interventions on adolescents’ negative affect in different cultural contexts.

## Data Availability

The original contributions presented in the study are included in the article/**Supplementary Material**. Further inquiries can be directed to the corresponding author.

## References

[1] Gellman MD . Encyclopedia of behavioral medicine. Berlin, Germany: Springer (2020).

[2] Oliveira Carvalho P Hülsdünker T Carson F . The impact of the COVID-19 lockdown on European students’ negative emotional symptoms: A systematic review and meta-analysis. Behav Sci. (2021) 12:3. doi: 10.3390/bs12010003 35049614 PMC8772797

[3] Wilson S Hicks BM Foster KT McGue M Iacono WG . Age of onset and course of major depressive disorder: associations with psychosocial functioning outcomes in adulthood. Psychol Med. (2015) 45:505–14. doi: 10.1017/s0033291714001640 PMC4289461 25007761

[4] Thapar A Collishaw S Pine DS Thapar AK . Depression in adolescence. Lancet. (2012) 379:1056–67. doi: 10.1016/s0140-6736(11)60871-4 PMC3488279 22305766

[5] Gore FM Bloem PJ Patton GC Ferguson J Joseph V Coffey C . Global burden of disease in young people aged 10–24 years: a systematic analysis. Lancet. (2011) 377:2093–102. doi: 10.1016/S0140-6736(11)60512-6 21652063

[6] Kessler RC Angermeyer M Anthony JC RDEG Demyttenaere K Gasquet I . Lifetime prevalence and age-of-onset distributions of mental disorders in the World Health Organization’s World Mental Health Survey Initiative. World Psychiatry. (2007) 6:168–76. PMC2174588 18188442

[7] Kessler RC Berglund P Demler O Jin R Merikangas KR Walters EE . Lifetime prevalence and age-of-onset distributions of DSM-IV disorders in the National Comorbidity Survey Replication. Arch Gen Psychiatry. (2005) 62:593–602. doi: 10.1001/archpsyc.62.6.593 15939837

[8] Merikangas KR He Burstein J-p M Swanson SA Avenevoli S Cui L . Lifetime prevalence of mental disorders in US adolescents: results from the National Comorbidity Survey Replication–Adolescent Supplement (NCS-A). J Am Acad Child Adolesc Psychiatry. (2010) 49:980–9. doi: 10.1016/j.jaac.2010.05.017 PMC2946114 20855043

[9] Chiu A Falk A Walkup JT . Anxiety disorders among children and adolescents. Focus. (2016) 14:26–33. doi: 10.1176/appi.focus.20150029 31975791 PMC6524434

[10] Beddington J Cooper CL Field J Goswami U Huppert FA Jenkins R . The mental wealth of nations. Nature. (2008) 455:1057–60. doi: 10.1038/4551057a 18948946

[11] Schmithorst VJ Yuan W . White matter development during adolescence as shown by diffusion MRI. Brain Cogn. (2010) 72:16–25. doi: 10.1016/j.bandc.2009.06.005 19628324

[12] Lenroot RK Giedd JN . Brain development in children and adolescents: insights from anatomical magnetic resonance imaging. Neurosci Biobehav Rev. (2006) 30:718–29. doi: 10.1016/j.neubiorev.2006.06.001 16887188

[13] Nelson SC Kling J Wängqvist M Frisén A Syed M . Identity and the body: Trajectories of body esteem from adolescence to emerging adulthood. Dev Psychol. (2018) 54:1159. doi: 10.1037/dev0000435 29620385

[14] Beauchamp MR Puterman E Lubans DR . Physical inactivity and mental health in late adolescence. JAMA Psychiatry. (2018) 75:543–4. doi: 10.1001/jamapsychiatry.2018.0385 29710114

[15] Boone SD Brausch AM . Physical activity, exercise motivations, depression, and nonsuicidal self-injury in youth. Suicide Life-Threatening Behav. (2016) 46:625–33. doi: 10.1111/sltb.2016.46.issue-5 26970091

[16] Blakemore S-J . The social brain in adolescence. Nat Rev Neurosci. (2008) 9:267–77. doi: 10.1038/nrn2353 18354399

[17] Burnett S Sebastian C Kadosh KC Blakemore S-J . The social brain in adolescence: evidence from functional magnetic resonance imaging and behavioural studies. Neurosci Biobehav Rev. (2011) 35:1654–64. doi: 10.1016/j.neubiorev.2010.10.011 PMC4538788 21036192

[18] Somerville LH Hare T Casey B . Frontostriatal maturation predicts cognitive control failure to appetitive cues in adolescents. J Cogn Neurosci. (2011) 23:2123–34. doi: 10.1162/jocn.2010.21572 PMC3131482 20809855

[19] Rice F Riglin L Lomax T Souter E Potter R Smith D . Adolescent and adult differences in major depression symptom profiles. J Affect Disord. (2019) 243:175–81. doi: 10.1016/j.jad.2018.09.015 30243197

[20] Johnco C Rapee RM . Depression literacy and stigma influence how parents perceive and respond to adolescent depressive symptoms. J Affect Disord. (2018) 241:599–607. doi: 10.1016/j.jad.2018.08.062 30172212

[21] Harder VS Barry SE French S Consigli AB Frankowski BL . Improving adolescent depression screening in pediatric primary care. Acad Pediatr. (2019) 19:925–33. doi: 10.1016/j.acap.2019.02.014 30858080

[22] Mendelson T Tandon SD . Prevention of depression in childhood and adolescence. Child Adolesc Psychiatr Clinics. (2016) 25:201–18. doi: 10.1016/j.chc.2015.11.005 26980124

[23] Xavier A Pinto-Gouveia J Cunha M Carvalho S . Self-criticism and depressive symptoms mediate the relationship between emotional experiences with family and peers and self-injury in adolescence. J Psychol. (2016) 150:1046–61. doi: 10.1080/00223980.2016.1235538 27715606

[24] Fluharty M Taylor AE Grabski M Munafò MR . The association of cigarette smoking with depression and anxiety: a systematic review. Nicotine Tobacco Res. (2016) 19:3–13. doi: 10.1093/ntr/ntw140 PMC5157710 27199385

[25] Sebena R El Ansari W Stock C Orosova O Mikolajczyk RT . Are perceived stress, depressive symptoms and religiosity associated with alcohol consumption? A survey of freshmen university students across five European countries. Subst Abuse treatment prevention Policy. (2012) 7:1–10. doi: 10.1186/1747-597X-7-21 PMC3395565 22640549

[26] Hofstra MB van der Ende J Verhulst FC . Child and adolescent problems predict DSM-IV disorders in adulthood: a 14-year follow-up of a Dutch epidemiological sample. J Am Acad Child Adolesc Psychiatry. (2002) 41:182–9. doi: 10.1097/00004583-200202000-00012 11837408

[27] Wang X Cai Z-D Jiang W-T Fang Y-Y Sun W-X Wang X . Systematic review and meta-analysis of the effects of exercise on depression in adolescents. Child Adolesc Psychiatry Ment Health. (2022) 16:16. doi: 10.1186/s13034-022-00453-2 35227300 PMC8886903

[28] White RL Olson R Parker PD Astell-Burt T Lonsdale C . A qualitative investigation of the perceived influence of adolescents’ motivation on relationships between domain-specific physical activity and positive and negative affect. Ment Health Phys Activity. (2018) 14:113–20. doi: 10.1016/j.mhpa.2018.03.002

[29] Kruk M Zarychta K Horodyska K Boberska M Scholz U Radtke T . What comes first, negative emotions, positive emotions, or moderate-to-vigorous physical activity? Ment Health Phys Activity. (2019) 16:38–42. doi: 10.1016/j.mhpa.2019.03.002

[30] Voss MW Nagamatsu LS Liu-Ambrose T Kramer AF . Exercise, brain, and cognition across the life span. J Appl Physiol. (2011) 111:1505–13. doi: 10.1152/japplphysiol.00210.2011 PMC3220305 21527670

[31] Zhu X Haegele JA Healy S . Movement and mental health: Behavioral correlates of anxiety and depression among children of 6–17 years old in the US. Ment Health Phys Activity. (2019) 16:60–5. doi: 10.1016/j.mhpa.2019.04.002

[32] Lu S Cheval B Yu Q Hossain MM Chen S-T Taylor A . Associations of 24-hour movement behavior with depressive symptoms and anxiety in children: cross-sectional findings from a Chinese sample. Healthcare. (2021) 9(11):1532. doi: 10.3390/healthcare9111532 34828578 PMC8620023

[33] Moher D Liberati A Tetzlaff J Altman DG PRISMA Group* t . Preferred reporting items for systematic reviews and meta-analyses: the PRISMA statement. Ann Internal Med. (2009) 151:264–9. doi: 10.7326/0003-4819-151-4-200908180-00135 PMC3090117 21603045

[34] Higgins JP Green S . Cochrane handbook for systematic reviews of interventions. (2008). doi: 10.1002/9780470712184

[35] Collaboration, C . Review manager (RevMan) version 5.3. Copenhagen: The Nordic Cochrane Centre (2014).

[36] Cohen J . Statistical power analysis for the behavioral sciences. New York: Routledge (2013).

[37] Moher D Shamseer L Clarke M Ghersi D Liberati A Petticrew M . Preferred reporting items for systematic review and meta-analysis protocols (PRISMA-P) 2015 statement. Systematic Rev. (2015) 4:1–9. doi: 10.1186/2046-4053-4-1 PMC4320440 25554246

[38] Cumpston M Li T Page MJ Chandler J Welch VA Higgins JP . Updated guidance for trusted systematic reviews: a new edition of the Cochrane Handbook for Systematic Reviews of Interventions. Cochrane Database Syst Rev. (2019) 10:Ed000142. doi: 10.1002/14651858.Ed000142 31643080 PMC10284251

[39] Ahmed KR Horwood S Khan A . Effects of a school-based physical activity intervention on adolescents’ Mental health: a cluster randomized controlled trial. J Phys activity Health. (2023) 20:1102–8. doi: 10.1123/jpah.2023-0062 37611913

[40] Carter T Guo B Turner D Morres I Khalil E Brighton E . Preferred intensity exercise for adolescents receiving treatment for depression: a pragmatic randomised controlled trial. BMC Psychiatry. (2015) 15:247. doi: 10.1186/s12888-015-0638-z 26467764 PMC4605143

[41] Crews DJ Lochbaum MR Landers DM . Aerobic physical activity effects on psychological well-being in low-income Hispanic children. Percept Mot Skills. (2004) 98:319–24. doi: 10.2466/pms.98.1.319-324 15058892

[42] Goldfield GS Kenny GP Alberga AS Prud'homme D Hadjiyannakis S Gougeon R . Effects of aerobic training, resistance training, or both on psychological health in adolescents with obesity: the HEARTY randomized controlled trial. J Of Consulting And Clin Psychol. (2015) 83:1123–35. doi: 10.1037/ccp0000038 26322787

[43] Hughes CW Barnes S Barnes C Defina LF Nakonezny P Emslie GJ . Depressed Adolescents Treated with Exercise (DATE): A pilot randomized controlled trial to test feasibility and establish preliminary effect sizes. Ment Health Phys Act. (2013) 6:119–31. doi: 10.1016/j.mhpa.2013.06.006 PMC3827851 24244220

[44] Khalsa SBS Hickey-Schultz L Cohen D Steiner N Cope S . Evaluation of the mental health benefits of yoga in a secondary school: A preliminary randomized controlled trial. J Of Behav Health Serv Res. (2012) 39:80–90. doi: 10.1007/s11414-011-9249-8 21647811

[45] Lima RA de Barros MVG Bezerra J Dos Santos SJ Monducci E Rodriguez-Ayllon M . Universal school-based intervention targeting depressive symptoms in adolescents: a cluster randomized trial. Scandinavian J Med Sci sports. (2022) 32:622–31. doi: 10.1111/sms.14115 34923679

[46] Melnyk BM Jacobson D Kelly SA Belyea MJ Shaibi GQ Small L . Twelve-month effects of the COPE healthy lifestyles TEEN program on overweight and depressive symptoms in high school adolescents. J school Health. (2015) 85:861–70. doi: 10.1111/josh.12342 PMC5117907 26522175

[47] Nabkasorn C Miyai N Sootmongkol A Junprasert S Yamamoto H Arita M . Effects of physical exercise on depression, neuroendocrine stress hormones and physiological fitness in adolescent females with depressive symptoms. Eur J Public Health. (2006) 16:179–84. doi: 10.1093/eurpub/cki159 16126743

[48] Noggle JJ Steiner NJ Minami T Khalsa SB . Benefits of yoga for psychosocial well-being in a US high school curriculum: a preliminary randomized controlled trial. J Dev Behav Pediatr. (2012) 33:193–201. doi: 10.1097/DBP.0b013e31824afdc4 22343481

[49] Norris R Carroll D Cochrane R . The effects of physical activity and exercise training on psychological stress and well-being in an adolescent population. J Psychosomatic Res. (1992) 36:55–65. doi: 10.1016/0022-3999(92)90114-H 1538350

[50] Philippot A Dubois V Lambrechts K Grogna D Robert A Jonckheer U . Impact of physical exercise on depression and anxiety in adolescent inpatients: a randomized controlled trial. J Affect Disord. (2022) 301:145–53. doi: 10.1016/j.jad.2022.01.011 35007642

[51] Chung JOK Li WHC Ho KY Lam KKW Cheung AT Ho LLK . Adventure-based training to enhance resilience and reduce depressive symptoms among juveniles: A randomized controlled trial. Res In Nurs Health. (2021) 44:438–48. doi: 10.1002/nur.22127 33754400

[52] Jeong YJ Hong SC Lee MS Park MC Kim YK Suh CM . Dance movement therapy improves emotional responses and modulates neurohormones in adolescents with mild depression. Int J Neurosci. (2005) 115:1711–20. doi: 10.1080/00207450590958574 16287635

[53] Roshan VD Pourasghar M Mohammadian Z . The efficacy of intermittent walking in water on the rate of MHPG sulfate and the severity of depression. Iranian J Psychiatry Behav Sci. (2011) 5:26–31. PMC3939959 24644444

[54] Olson RD Vaux-Bjerke A Quam JB Piercy KL Troiano RP George SM . Physical activity guidelines for Americans. In: NADAR! SWIMMING MAGAZINE-Periódico científico em esportes e fitness aquático-natação, pólo aquático, nado sincronizado, saltos ornamentais, travessias aquáticas. Washington: United States Department of Health and Human Services (2023).

[55] Sallis JF Prochaska JJ Taylor WC . A review of correlates of physical activity of children and adolescents. Med Sci sports Exercise. (2000) 32:963–75. doi: 10.1097/00005768-200005000-00014 10795788

[56] Bertele S Heitland I Fraccarollo D Stapel B Bauersachs J Westhoff-Bleck M . Behavioral pathway to a broken heart: the link between adverse childhood experiences, depression, physical exercise and cardiovascular health. Front Psychiatry. (2022) 13:1002143. doi: 10.3389/fpsyt.2022.1002143 36304562 PMC9595725

[57] Morgan WP . Affective beneficence of vigorous physical activity. Med Sci sports Exercise. (1985) 17:94–100. doi: 10.1249/00005768-198502000-00015 3157040

[58] Yeung RR . The acute effects of exercise on mood state. J psychosomatic Res. (1996) 40:123–41. doi: 10.1016/0022-3999(95)00554-4 8778396

[59] Tao R Liang S Bao C Zhang J Zhang C . Relationships between physical activity, sedentary behavior and anxiety in chinese children with visual impairment: A cross-lagged analysis. J Dev Phys Disabil. (2023) 35:759–73. doi: 10.1007/s10882-022-09879-0

[60] Gehricke J-G Lowery LA Alejo SD Dawson M Chan J Parker RA . The effects of a physical exercise program, LEGOR and Minecraft activities on anxiety in underserved children with autism spectrum disorder. Res Autism Spectr Disord. (2022) 97:102005. doi: 10.1016/j.rasd.2022.102005

[61] Rodriguez-Ayllon M Cadenas-Sánchez C Estévez-López F Muñoz NE Mora-Gonzalez J Migueles JH . Role of physical activity and sedentary behavior in the mental health of preschoolers, children and adolescents: a systematic review and meta-analysis. Sports Med. (2019) 49:1383–410. doi: 10.1007/s40279-019-01099-5 30993594

[62] Szabo A . Acute psychological benefits of exercise: Reconsideration of the placebo effect. J Ment Health. (2013) 22:449–55. doi: 10.3109/09638237.2012.734657 23324013

[63] Kvam S Kleppe CL Nordhus IH Hovland A . Exercise as a treatment for depression: a meta-analysis. J Affect Disord. (2016) 202:67–86. doi: 10.1016/j.jad.2016.03.063 27253219

[64] Song J Liu Z-Z Huang J Wu J-S Tao J . Effects of aerobic exercise, traditional Chinese exercises, and meditation on depressive symptoms of college student: A meta-analysis of randomized controlled trials. Medicine. (2021) 100:e23819. doi: 10.1097/MD.0000000000023819 33429742 PMC7793414

[65] Petruzzello SJ Landers DM Hatfield BD Kubitz KA Salazar W . A meta-analysis on the anxiety-reducing effects of acute and chronic exercise: Outcomes and mechanisms. Sports Med. (1991) 11:143–82. doi: 10.2165/00007256-199111030-00002 1828608

[66] Sharifi M Hamedinia M Hosseini-Kakhak S . The effect of an exhaustive aerobic, anaerobic and resistance exercise on serotonin, beta-endorphin and BDnf in students. Phys Educ students. (2018) 5:272–7. doi: 10.15561/20755279.2018.0507

[67] Lampinen P Heikkinen E . Reduced mobility and physical activity as predictors of depressive symptoms among community-dwelling older adults: an eight-year follow-up study. Aging Clin Exp Res. (2003) 15:205–11. doi: 10.1007/BF03324501 14582683

[68] Chen H Tsai C Wu Y Lin K Lin C . Randomised controlled trial on the effectiveness of home-based walking exercise on anxiety, depression and cancer-related symptoms in patients with lung cancer. Br J Cancer. (2015) 112:438–45. doi: 10.1038/bjc.2014.612 PMC4453645 25490525

[69] Abedi P Nikkhah P Najar S . Effect of pedometer-based walking on depression, anxiety and insomnia among postmenopausal women. Climacteric. (2015) 18:841–5. doi: 10.3109/13697137.2015.1065246 26100101

[70] Werner-Seidler A Perry Y Calear AL Newby JM Christensen H . School-based depression and anxiety prevention programs for young people: A systematic review and meta-analysis. Clin Psychol Rev. (2017) 51:30–47. doi: 10.1016/j.cpr.2016.10.005 27821267

[71] Stockings E Degenhardt L Dobbins T Lee YY Erskine H Whiteford H . Preventing depression and anxiety in young people: a review of the joint efficacy of universal, selective and indicated prevention. psychol Med. (2016) 46:11–26. doi: 10.1017/S0033291715001725 26315536

[72] Singh B Olds T Curtis R Dumuid D Virgara R Watson A . Effectiveness of physical activity interventions for improving depression, anxiety and distress: an overview of systematic reviews. Br J sports Med. (2023) 57:1203–9. doi: 10.1136/bjsports-2022-106195 PMC10579187 36796860

[73] Chang Y-C Lu M-C Hu I-H Wu W-CI Hu SC . Effects of different amounts of exercise on preventing depressive symptoms in community-dwelling older adults: a prospective cohort study in Taiwan. BMJ Open. (2017) 7:e014256. doi: 10.1136/bmjopen-2016-014256 PMC5623457 28465305

[74] Paluska SA Schwenk TL . Physical activity and mental health: current concepts. Sports Med. (2000) 29:167–80. doi: 10.2165/00007256-200029030-00003 10739267

[75] Tremblay MS LeBlanc AG Janssen I Kho ME Hicks A Murumets K . Canadian sedentary behaviour guidelines for children and youth. Appl Physiology Nutrition Metab. (2011) 36:59–64. doi: 10.1139/H11-012 21326378

[76] Kersey RD . Anabolic-androgenic steroid use by private health club/gym athletes. J Strength Conditioning Res. (1993) 7:118–26.

[77] Hetrick SE Cox GR Merry SN . Treatment-resistant depression in adolescents: is the addition of cognitive behavioral therapy of benefit? Psychol Res Behav Manage. (2011) 4:97–112. doi: 10.2147/PRBM.S13780 PMC3218778 22114540

