# Supplementary material for: The influence of physical exercise on negative emotions in adolescents: a meta-analysis
Source: Front Psychiatry. 2024 Nov 12;15:1457931. doi: 10.3389/fpsyt.2024.1457931 (PMC11588725; doi:10.3389/fpsyt.2024.1457931)
Supplement: Supplementary file 1 [file DataSheet1.pdf]

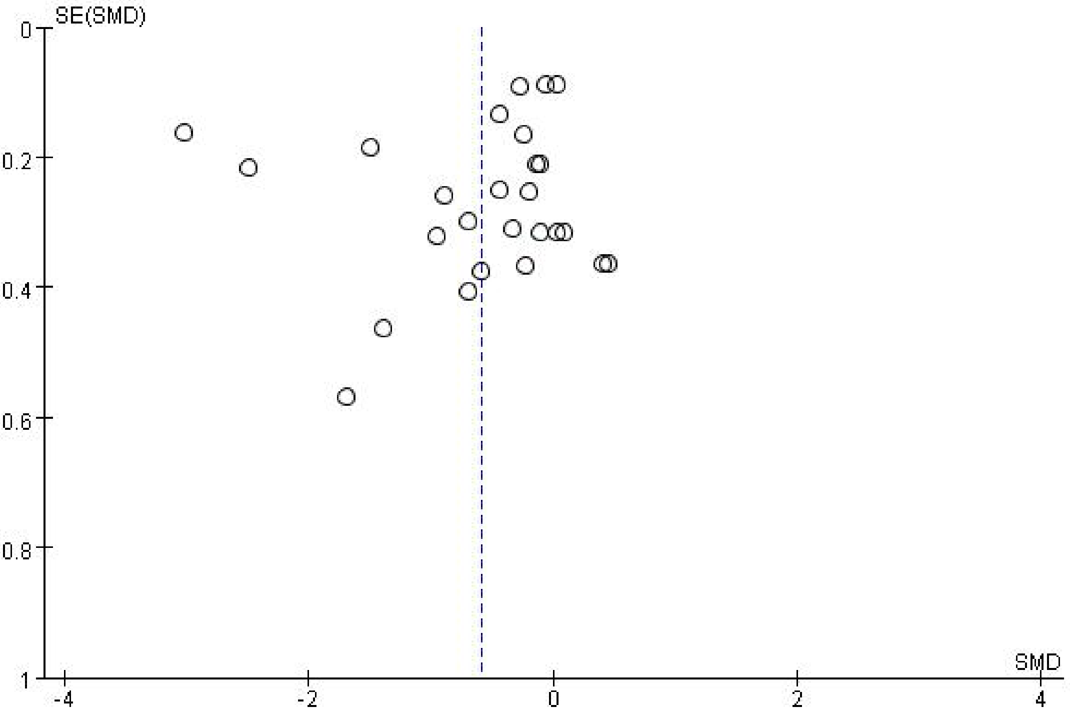

Supplementary Figure 1: Funnel plot publication bias in the relationship between PE and negative emotions in adolescents

	Experimental			Control				Std. Mean Difference
Study or Subgroup	Mean	SD	Total	Mean	SD	Total	Weight	IV, Random, 95% CI
Ahmed (2023)	6.85	2.1	160	13.5	2.3	160	4.2%	-3.01 [-3.33, -2.69]
Carter (2015)	23.8	10.7	36	25.7	8.5	28	3.9%	-0.19 [-0.69, 0.30]
Chung (2021)	18.2	6.2	115	20.7	5.2	113	4.2%	-0.44 [-0.70, -0.17]
Crews a (2004)	42.1	11.1	34	46.3	7.8	32	4.0%	-0.43 [-0.92, 0.06]
Crews b (2004)	9.5	9.5	34	18.7	11	32	3.9%	-0.89 [-1.39, -0.38]
Goldfield a (2015)	2.1	0.4	73	2.7	0.4	75	4.1%	-1.49 [-1.86, -1.13]
Goldfield b (2015)	1.7	0.4	78	2.7	0.4	75	4.0%	-2.49 [-2.91, -2.06]
Goldfield c (2015)	2.6	0.4	72	2.7	0.4	75	4.2%	-0.25 [-0.57, 0.08]
Hughes (2013)	24.1	5.8	14	28.3	5.98	12	3.5%	-0.69 [-1.49, 0.11]
Jeong (2005)	46.4	10.2	20	46.1	5.7	20	3.8%	0.04 [-0.58, 0.66]
Khalsa a (2012)	-0.48	3.8	67	-0.03	4.7	34	4.1%	-0.11 [-0.52, 0.30]
Khalsa b (2012)	-0.59	3.9	67	0.03	5.7	34	4.1%	-0.13 [-0.55, 0.28]
Lima a (2022)	17.3	11.2	308	17.9	11.4	218	4.3%	-0.05 [-0.23, 0.12]
Lima b (2022)	15.1	9.2	251	17.9	11.4	218	4.3%	-0.27 [-0.45, -0.09]
Lima c (2022)	18.3	11.5	289	17.9	11.4	218	4.3%	0.03 [-0.14, 0.21]
Melnyk (2015)	42.39	11.82	9	57.9	3.77	9	2.9%	-1.68 [-2.80, -0.57]
Nabkasorn (2006)	14.1	4.58	21	17.4	4.76	28	3.8%	-0.69 [-1.28, -0.11]
Noggle a (2012)	5.1	3.6	36	9.3	5.8	15	3.7%	-0.95 [-1.58, -0.32]
Noggle b (2012)	4.7	4.9	36	6.3	4.2	15	3.8%	-0.33 [-0.94, 0.27]
Norris a (1992)	4.21	2.08	14	6.25	4.14	16	3.6%	-0.59 [-1.33, 0.14]
Norris b (1992)	7.8	2.25	15	6.25	4.14	16	3.6%	0.45 [-0.27, 1.16]
Norris d (1992)	9.14	5.19	14	10.19	4.15	16	3.6%	-0.22 [-0.94, 0.50]
Norris e (1992)	11.6	2.17	15	10.19	4.15	16	3.6%	0.41 [-0.30, 1.12]
Philippot a (2022)	10.7	5.5	20	11.3	6.4	20	3.8%	-0.10 [-0.72, 0.52]
Philippot b (2022)	9.4	4.5	20	6.5	46	20	3.8%	0.09 [-0.53, 0.71]
Roshan (2011)	14.08	5.79	12	25.58	9.72	12	3.3%	-1.39 [-2.30, -0.48]
**Total (95% CI)**			**1830**			**1527**	**100.0%**	**-0.59 [-0.92, -0.26]**

Heterogeneity: $Tau^2 = 0.65$; $Chi^2 = 458.54$, df = 25 ($P < 0.00001$); $I^2 = 95\%$
Test for overall effect: $Z = 3.50$ ($P = 0.0005$)

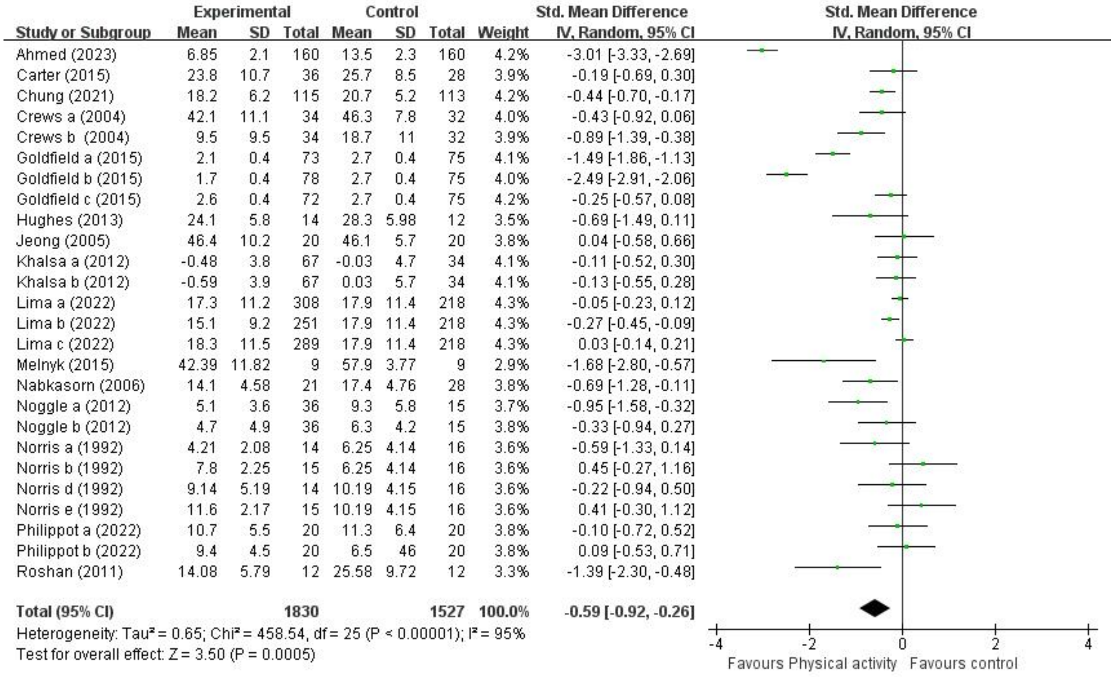

Supplementary Figure 2: Forest plot of a meta-analysis of the relationship between PE and negative emotions after the intervention
